# DeployFusion: A Deployable Monocular 3D Object Detection with Multi-Sensor Information Fusion in BEV for Edge Devices

**DOI:** 10.3390/s24217007

**Published:** 2024-10-31

**Authors:** Fei Huang, Shengshu Liu, Guangqian Zhang, Bingsen Hao, Yangkai Xiang, Kun Yuan

**Affiliations:** 1 China Road and Bridge Corporation, Beijing 100010, China; huangf@crbc.com (F.H.); liuss@crbc.com (S.L.); 2 Chongqing Seres Phoenix Intelligent Innovation Technology Co., Ltd., Chongqing 400039, China; gqzhang77@163.com; 3 School of Mechatronics and Vehicle Engineering, Chongqing Jiaotong University, Chongqing 400074, China; xiangyangkai@163.com (Y.X.); 18280447819@163.com (K.Y.)

**Keywords:** multi-sensor information fusion, 3D object detection, BEV, feature fusion, model deployment

## Abstract

To address the challenges of suboptimal remote detection and significant computational burden in existing multi-sensor information fusion 3D object detection methods, a novel approach based on Bird’s-Eye View (BEV) is proposed. This method utilizes an enhanced lightweight EdgeNeXt feature extraction network, incorporating residual branches to address network degradation caused by the excessive depth of STDA encoding blocks. Meantime, deformable convolution is used to expand the receptive field and reduce computational complexity. The feature fusion module constructs a two-stage fusion network to optimize the fusion and alignment of multi-sensor features. This network aligns image features to supplement environmental information with point cloud features, thereby obtaining the final BEV features. Additionally, a Transformer decoder that emphasizes global spatial cues is employed to process the BEV feature sequence, enabling precise detection of distant small objects. Experimental results demonstrate that this method surpasses the baseline network, with improvements of 4.5% in the NuScenes detection score and 5.5% in average precision for detection objects. Finally, the model is converted and accelerated using TensorRT tools for deployment on mobile devices, achieving an inference time of 138 ms per frame on the Jetson Orin NX embedded platform, thus enabling real-time 3D object detection.

## 1. Introduction

Three-dimensional object detection enables autonomous vehicles to accurately recognize their environments by identifying the position, category, and geometric information of both static and dynamic objects. This capability is crucial for optimal decision making and motion control, thereby enhancing driving safety and efficiency [1]. Lidar [2] and cameras serve as two primary sensors in the autonomous driving perception system, each capturing environmental information differently. Specifically, Lidar emits light beams of specific wavelengths and receives their reflected signals to generate point cloud data, precisely locating the spatial positions of objects and obtaining detailed geometric shapes. In contrast, cameras provide rich scene images containing dense semantic and texture information that serves as an important supplement to point cloud data. However, due to the inherent differences between Lidar and cameras, effectively integrating these two modalities of information remains a challenging issue in the current research field.

Traditional multi-sensor information fusion methods for 3D object detection, when constrained by conventional fields of view (FOV), are limited to a single source of environmental information due to the camera’s restricted vision. This limitation considerably impairs the system’s perception capabilities, rendering it susceptible to environmental factors, lighting conditions, and occlusions. Consequently, these methods face challenges in fulfilling the requirements of autonomous driving, particularly in providing accurate orientation and positional information within the surrounding 3D space. In contrast, multi-sensor information fusion 3D object detection methods under Bird’s-Eye View (BEV) [3] leverage the integration of multiple sensor data types to comprehensively and accurately acquire vehicle environmental information, and exhibit better detection performance for occluded objects.

Despite numerous research achievements in 3D object detection through multi-sensor information fusion, several challenges remain: (1) Effectively integrating multi-sensor feature information from LiDAR and cameras is crucial for enhancing long-range detection accuracy, given their inherent differences. (2) Although methods based on attention mechanisms have significantly improved accuracy, the substantial increase in computational complexity limits their deployment on mobile computing devices.

The contributions of this study are summarized as follows:

- We propose a lightweight and efficient image feature extraction network, EdgeNeXt_DCN, which integrates residual branches to prevent degradation in deep networks. By employing deformable convolutions, the network expands the receptive field while reducing computational load, achieving feature learning capabilities comparable to Swin-Transformer.
- A two-stage fusion network is constructed to align image features with point cloud features, supplementing environmental information. This optimization enhances the fusion and alignment of different modal features, resulting in accurate BEV features.
- Compared to the baseline model network, the NuScenes detection score and average object accuracy have been improved by 4.5% and 5.5%, respectively. Deployment on mobile edge devices shows an inference latency of approximately 138 milliseconds.

## 2. Related Work

### 2.1. 3D Object Detection via Multi-Sensor Information Fusion

Wang et al. [4] categorized multi-sensor fusion methods into pre-fusion, feature fusion, and post-fusion. Pre-fusion methods combine point cloud data with image semantic features [5], but the substantial computational load made deployment challenging. Building on this, Meyer et al. [6] utilized convolutional neural networks to fuse voxelized point clouds with image features, achieving improved detection performance. Similarly, merging raw image pixels with voxelized point clouds [7] or directly integrating pseudo-point clouds generated from images with original point clouds [8] accomplishes 3D object detection tasks. However, due to significant information discrepancies between multi- sensor data, these methods’ performance is suboptimal. Post-fusion methods primarily focus on post-processing detection results from each modality. Pang et al. [9] combined detection candidate regions from two modalities to predict 3D object attributes. Gu et al. [10,11] fused multi-sensor detection structures using segmentation results for road detection. Braun et al. [12] projected 3D proposal boxes from the point cloud branch onto the 2D proposal boxes of the image branch to refine the detection results. However, traditional spatial-aware frameworks that primarily rely on late fusion strategies exhibit poor method robustness. Pandey et al. [13] argue that the interconnections among different data streams are often found at higher levels, and early fusion of multi-sensor data struggles to fully demonstrate the complementarity between modalities [14]. Recognizing distant small objects within a field of view is challenging, and effectively integrating multi-sensor feature information is key. However, current methods still have shortcomings in fusing modal feature information, limiting improvements in distant object detection accuracy. Fusion detection methods that use unified feature representations in BEV can better characterize the complementarity between modalities. Huang et al. [15,16] leveraged the global view characteristics of BEV, continuing with BEVDet to introduce BEVDet4D, which incorporates historical information for 3D object detection in 4D space. This method shows improvements in object position, orientation, and speed prediction, but the introduction of 4D information incurs high inference latency. Subsequently, Liu et al. [17,18] unified multi-sensor feature representations in BEV space, constructing a multi-task multi-sensor fusion framework. This approach, with its simple network structure and good detection performance, further propelled research on multi-sensor deep fusion methods. However, these methods experience a significant increase in computational complexity when calculating cross-feature layer attention, making them difficult to deploy on mobile edge devices.

### 2.2. Model Deployment

In the field of 3D object detection, the high computational complexity and large parameter count of deep learning models make it challenging to achieve real-time detection on vehicle-mounted mobile devices, posing a significant barrier to commercial applications. Xu et al. [19] successfully deployed YOLOv3-Promote on embedded devices through model restructuring, pruning, and semi-precision acceleration. Dai et al. [20] utilized TensorRT to optimize deep learning models, accelerating inference speed on embedded platforms and enhancing real-time performance. However, these methods primarily focus on 2D object detection and have not been validated in 3D object detection, with input image resolutions being relatively low. Tang et al. [21] employed C++, TensorRT, Float16 quantization, and oneTBB on edge computing platforms, significantly enhancing method performance. Zhang et al. [22] improved the processing capability of embedded devices for point cloud data through model optimization, INT8 quantization techniques, and hardware acceleration. However, unlike the embedded devices used in this paper, the aforementioned methods were deployed on the higher-performance Jetson AGX Orin edge computing device.

## 3. Methods

### 3.1. Design of the Overall Network

**Figure 1:**
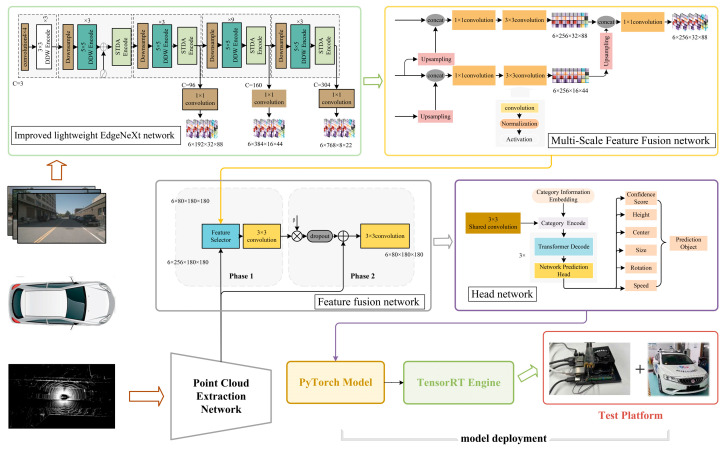
Overall framework of the network. DeployFusion introduces an improved EdgeNeXt feature extraction network, using residual branches to address degradation and deformable convolutions to increase the receptive field and reduce complexity. The feature fusion module aligns image and point cloud features to generate optimized BEV features. A Transformer decoder is used to process the sequence of BEV features, enabling accurate identification of small distant objects.

The proposed network framework is illustrated in Figure 1. The visual feature extraction network utilizes an enhanced, lightweight EdgeNeXt network (EdgeNeXt_DCN) that incorporates residual branches to mitigate network degradation caused by excessive STDA encoding block depth. Additionally, it substitutes depth-wise convolutions with deformable convolutions to broaden the receptive field and decrease computational complexity. The multi-scale feature fusion network effectively handles scale variations and enhances model generalization by merging multi-scale details and features. Additionally, the feature fusion module incorporates a residual fusion structure and learnable adjustment parameters to enhance multi-sensor feature fusion and alignment. The detection head network is designed with a three-layer attention decoder that uses self-attention to decode the BEV feature sequence, enabling precise detection of distant small objects.

### 3.2. Improvement of Backbone Network

Traditional convolutional neural networks (CNNs) are limited in their ability to capture global feature interactions. Conversely, Vision Transformers (ViTs) offer enhanced interaction capabilities but are challenging to deploy on resource-constrained edge devices due to their high computational costs [23]. The lightweight EdgeNeXt_DCN architecture is used as the foundation for visual feature extraction. This network integrates a residual branch to address degradation caused by increased STDA-encoding block depth. Additionally, by replacing traditional depth-wise separable convolutions (DWC) with deformable convolutions, we aim to expand the receptive field while reducing computational complexity.

The EdgeNeXt_DCN network is subdivided into four main parts. In the initial stage, the input image undergoes downsampling through a 4 × 4 convolutional layer, followed by a 3 × 3 depth-wise separable convolution (DDW) encoding block to extract image features, resulting in a feature map with a resolution of 1/8. In the second stage, the introduction of positional encoding significantly enhances the capture of positional relationships between features, facilitating the extraction of key features. Subsequently, in the network design of the third, fourth, and fifth stages, feature maps with different depths of 96, 160, and 304 channels are, respectively, used as outputs, aiming to provide multi-scale image features for the feature fusion process. The downsampling module of the network consists of a set of 2 × 2 convolutional layers and batch normalization layers, while the 1 × 1 convolutional layer is responsible for adjusting the number of channels.

**Figure 2:**
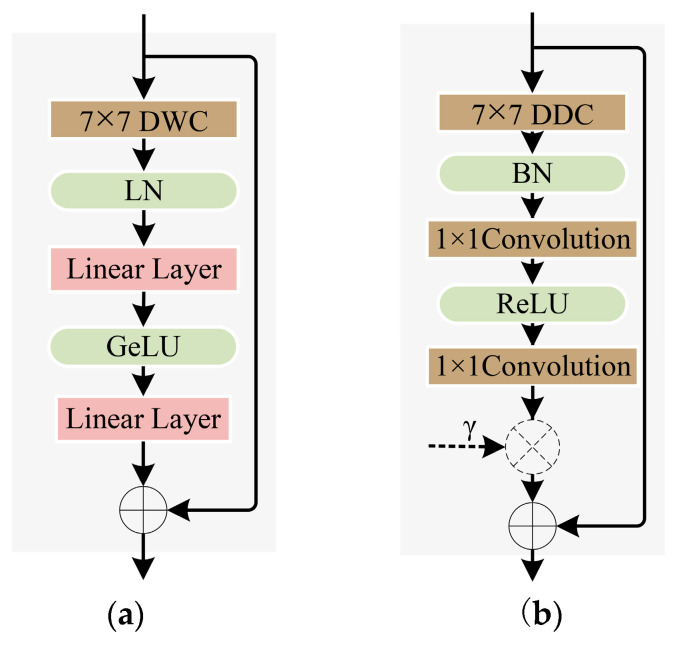
Comparison of convolutional encoding block. (**a**) DW Encode. (**b**) DDW Encode.

EdgeNeXt_DCN utilizes a depth-wise separable convolution (DWC) structure to construct the DW encoding block, which consists of a 7 × 7 depth-wise separable convolution and two linear layers. As shown in Figure 2, to expand the receptive field and reduce computational costs, deformable convolutions are used to replace the DWC within the DW encoding block, resulting in a deformable depth-wise separable convolution block, referred to as the DDW block. Additionally, learnable parameters γ are introduced based on the depth of the network, aiming to help the network effectively capture key features and thereby enhance its expressive capability. The proposed DDW encoding block is not only computationally efficient, but also capable of effectively capturing multi-scale object features in images.

**Figure 3:**
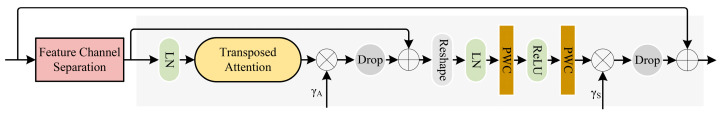
Feature channel separation attention.

As shown in Figure 3, the Split-Depth Transpose Attention Encoder Block (STDA) consists of a concatenation of feature channel separation attention and transpose attention. To address the issue of network degradation caused by excessive network depth within the STDA coding block, residual branches were added for fusion at both the structural output position and the transpose attention output position. Subsequently, learnable parameters and drop layers are incorporated before the two residual structures to adjust the feature map parameters during the fusion process, thereby enhancing the robustness of the network. The calculation formula is as follows:

(1) $$x=\gamma_{A}\cdot f_{\mathrm{Attn}}(K,Q,V)+x_{0}$$

(2) $$\overset{⌢}{x}=\gamma_{s}\cdot f_{\mathrm{ddc}}(C_{\mathrm{pwc}}(C_{\mathrm{pwc}}(x))+x_{0}$$

where x represents network input; $\overset{⌢}{x}$ denotes network output; $x_{0}$ represents the intermediate variable; $\gamma_{A}$ and $\gamma_{s}$ are learnable parameters; $f_{\mathrm{Attn}}$ and $f_{\mathrm{ddc}}$, respectively, denote transposed attention and feature channel classification calculations; $C_{\mathrm{pwc}}$ is the PWC network module computation.

**Figure 4:**
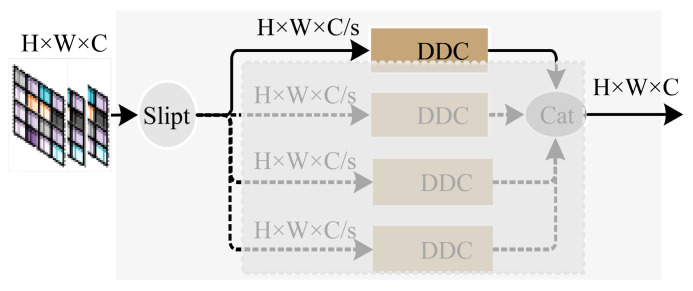
Feature channel separation attention.

Among them, the feature channel separation attention divides the feature map along the channel dimension, aiming to enable the network to learn more scale-specific feature information (as shown in Figure 4). Depending on the depth of the network, different segmentation scales *s* are employed to adjust the channel dimension within the branches, and the segmented features are concatenated at the backend. The channel separation attention promotes interaction between different channel features by encoding them, which facilitates targeted learning of key features and enhances the network’s representational capability.

**Figure 5:**
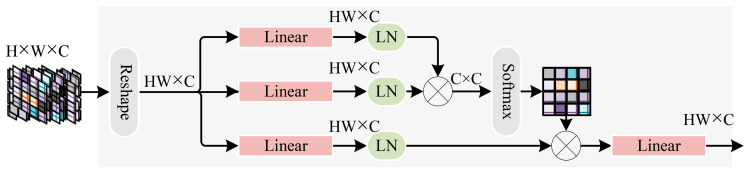
Transposed attention.

To reduce the computational complexity of the self-attention mechanism, transposed attention employs multi-head self-attention calculations across the channel dimension to obtain attention maps between feature channels (as shown in Figure 5). Subsequently, three linear layers are used to derive three feature sequences: Query (Q), Key (K), and Value (V) from the input features, each with a sequence dimension of HW×C. The traditional attention mechanism computes the dot product between Q and $K^{T}$, resulting in an attention matrix of dimension HW×HW. In contrast, transposed attention calculates the dot product between $Q^{T}$ and K across the channel dimension, yielding an attention matrix of dimension C×C. Compared to the traditional attention mechanism, transposed attention significantly reduces computational complexity, making it more suitable for deployment on mobile edge devices. The calculation formula is as follows:

(3) $$Q=W_{Q}Y$$

(4) $$K=W_{K}Y$$

(5) $$V=W_{V}Y$$

(6) $$\mathrm{Score}=V\cdot \mathrm{Softmax}(Q^{T}\cdot K)$$

where $W_{Q}$, $W_{K}$, and $W_{V}$ represent the network weights of *Q*, *K*, and *V* sequences, respectively. Score denotes the weight score.

### 3.3. Deformable Convolution

**Figure 6:**
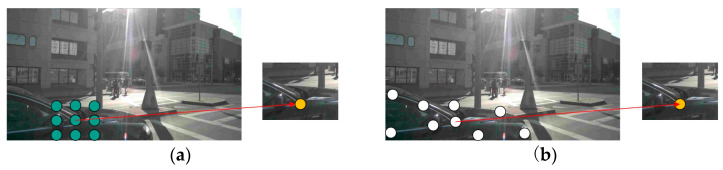
Comparison of standard and variable convolution kernels in receptive field regions. (**a**) Receptive field area of standard convolutional kernel. (**b**) Receptive field area of deformable convolutional kernel.

Traditional convolutional kernels have fixed shapes, which limits their ability to process objects with complex shapes or large feature variations. Deformable convolution with learnable offsets [24] is employed to adjust the positions of local feature points and the receptive field range, thereby enhancing adaptability to various shapes and features, as shown in Figure 6. Among them, green circles represent standard convolutional kernel features, while white circles represent deformable convolutional kernel features. The learnable offsets can adjust the shape of the convolutional kernel, allowing for more precise feature capture, particularly of object contours based on learned parameters. This enables the receptive field to adapt to the movement and scaling of the object. EdgeNeXt_DCN replaces traditional depth-wise separable convolution (DWC) with deformable convolution, aiming to expand the receptive field while reducing computational complexity.

The traditional convolution structure formula is as follows:

(7) R={(−1,−1),(−1,0),…,(0,1),(1,1)}

(8) $$y(p_{0})=\sum_{p_{n}\in R}w(p_{n})\cdot x(p_{0}+p_{n})$$

where w represents the weight. After introducing the learnable offset $\Delta p_{n}$, the convolution formula becomes:

(9) $$y(p_{0})=\sum_{p_{n}\in R}w(p_{n})\cdot x(p_{0}+p_{n}+\Delta p_{n})$$

Since the calculated offsets are often floating-point numbers, interpolation is required to determine the offset positions on the feature map. The formula is as follows:

(10) $$x(p_{0}+p_{n}+\Delta p_{n})=\sum_{q}G(q,p_{0}+p_{n}+\Delta p_{n})\cdot x(q)$$

where R represents the convolution range; $p_{0}$ denotes the point in the feature map corresponding to the center of the convolution kernel; $p_{n}$ is the sampling point of $p_{0}$ within the convolution range R; x(q) indicates the values of all integer-position points on the feature map; G refers to the bilinear interpolation method.

### 3.4. Improved Multi-Scale Feature Fusion Network

As illustrated in Figure 1, the multiscale feature fusion network adopts the structure of FPN [25] for feature integration, effectively enhancing the adaptability to scale variations and the generalization capability of the model by consolidating detailed information across different scales. In this architecture, features of the 8 × 22 scale undergo bilinear interpolation for feature sampling, which are then concatenated with feature maps of the 16 × 44 scale. Subsequently, the concatenated feature maps are fused using 1 × 1 and 3 × 3 convolutional layers. Ultimately, the fused features from the two branches, which are of sizes 256 × 32 × 88 and 256 × 16 × 44, respectively, are concatenated and passed through a 1 × 1 convolutional layer for adjustment of the feature channels.

The aforementioned structure is designed to capture multi-scale features within the scene. By employing deformable convolution, this approach enhances the network’s generalization capabilities and reduces computational complexity. Additionally, transposed attention operations are performed in the channel dimension, allowing the network to obtain richer feature representations at a lower computational cost.

### 3.5. Improved Feature Fusion Network

Due to the inherent spatial characteristics of point cloud data, voxel processing transforms the data into a spatial feature with a BEV representation through a multi-layer voxel feature encoder. This representation is then fused with camera modality features. The feature extraction network for LiDAR utilizes SECOND. Although the original BEVFusion network reduces computational demands through simple fusion methods such as Conv and Add, it struggles to efficiently align the features of different modalities. Consequently, within the unified BEV space, the alignment efficiency of the two modality features is low, leading to insufficient information fusion.

To address the challenge of aligning features from different modalities, a two-stage fusion network is proposed, which integrates diverse modal features through convolution and residual structures. The feature fusion process is illustrated in Figure 1. In the first stage, the network initially fuses two modal features, and in the second stage, it adjusts these fused features to align with point cloud characteristics. Specifically, the first stage employs a feature selector to randomly select feature proportions and uses 3 × 3 convolutional layers for fusion. The second stage optimizes the fused features through learnable adjustment parameters and employs dropout layers to reduce conflicts in inter-modal fusion. Finally, through a residual structure, the features processed by dropout are aligned with point cloud features to obtain the final BEV features. This process, based on point cloud features, efficiently aligns image features to supplement environmental information for point cloud features. Consequently, the feature fusion network effectively enhances the fusion and alignment of different modal features under the BEV.

### 3.6. Improved Detection Head Network

To achieve precise detection of distant small objects, a Transformer decoder emphasizing global spatial cues is employed to process the object sequence of BEV features. The detection head network architecture is illustrated in Figure 1, where the modality fusion features are decoded by the SECOND network and then fed into the detection head network for inference. The detection head incorporates three Transformer decoder layers, utilizing self-attention to decode the object sequences in the BEV features. Specifically, after the shared convolutional layers extract the point cloud features, these features serve as the Q, K, and V. They then undergo interaction and querying through multiple Transformer decoder layers and, combined with the heatmap scores, generate the final predicted objects.

To efficiently focus on category prediction, the FocalLoss and GaussianFocalLoss functions are employed to calculate the category prediction loss and the heatmap loss, respectively. The formulas for these calculations are as follows:

(11) $$L=L_{\mathrm{FL}}(x_{\mathrm{cls}})+L_{\mathrm{GFL}}(x_{\mathrm{heatmap}})+L_{L1}(x_{\mathrm{bbox}})$$

where L represents the overall loss function of the network; $L_{\mathrm{FL}}$, $L_{\mathrm{GFL}}$, and $L_{L1}$, respectively, denote the FocalLoss, GaussianFocalLoss, and L1 loss functions; and $x_{\mathrm{cls}}$, $x_{\mathrm{heatmap}}$, and $x_{\mathrm{bbox}}$ represent the predicted values of categories, heat maps, and bounding boxes during the training process.

## 4. Experiment and Result Analysis

### 4.1. Experiments Settings

This study examined the impact of image branch feature extraction networks on the performance of detection networks, using the BEVFusion model with Swin-Transformer as the image feature extraction network and the evaluation benchmark. During the training process, experiments were conducted on a workstation equipped with an Nvidia GeForce RTX4090 GPU. The software tools involved primarily included deep learning development libraries such as PyTorch, CUDA, and CUDNN, as well as the OpenCV image processing library. Based on the nuScenes V1.0-mini dataset, preliminary trials were conducted on various network structures to delve into and analyze the specific impacts of each network structure on the model detection performance.

### 4.2. Comparative Experiment of Feature Extraction Network

**Figure 7:**
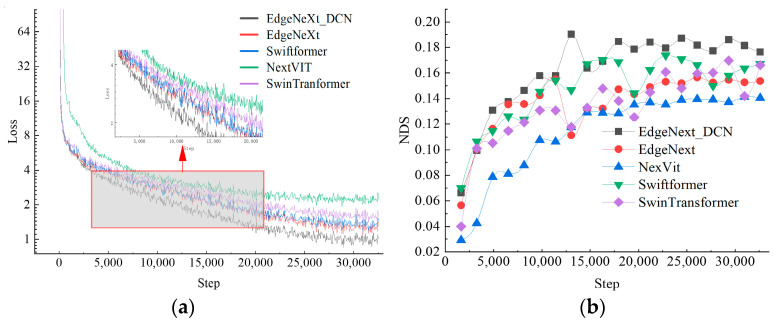
Experimental results of dynamic loss and NDS. (**a**) Dynamic loss graph. (**b**) Dynamic NDS score graph.

Under the BEV fusion framework, EdgeNeXt_DCN was tested with typical lightweight feature extraction networks such as NextVIT, Swiftformer, and EdgeNeXt, with the results shown in Figure 7.

The reduction in loss values often indicates that the method network has acquired effective feature representations, with its decline and convergence reflecting the method’s ability to capture data features and its stability. Compared to other networks, the EdgeNeXt_DCN network converges faster and is easier to stabilize, demonstrating that the method under this network can reach a stable state more rapidly. EdgeNeXt employs batch normalization for network normalization, and its test performance is comparable to that of the benchmark network. Notably, using Swiftformer and EdgeNeXt_DCN as feature extraction networks, their comprehensive detection metric, DNS, significantly outperforms the test benchmark. The EdgeNeXt_DCN network increases the NDS to 0.19 at 13,000 steps, representing an improvement of 0.07 and 0.08 over the Swin-Transformer and the original EdgeNeXt network, respectively. This demonstrates its capability to enhance network learning and detection performance. In contrast, the NextVIT structure provides a smaller improvement in detection method performance, slightly below the benchmark.

**Figure 8:**
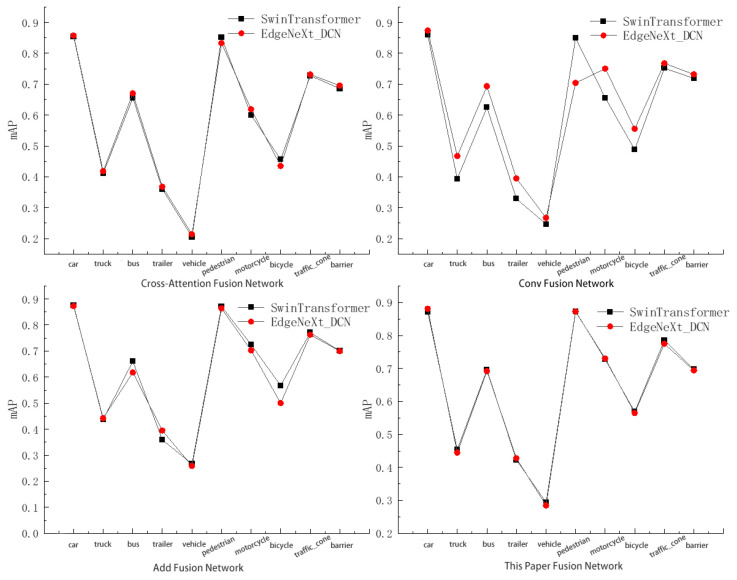
Comparison of EdgeNeXt_DCN with other fusion networks of inference results.

A comparison was made between the Swin-Transformer and EdgeNeXt_DCN networks under different fusion networks in the context of multi-sensor object detection methods, with the results depicted in Figure 8. The results demonstrate that the EdgeNeXt_DCN network exhibits strong feature extraction capabilities in both cross-attention fusion and Conv fusion scenarios. Consequently, the EdgeNeXt_DCN network achieves higher detection accuracy across most categories. In contrast, under Add fusion and the feature fusion network designed in this study, EdgeNeXt_DCN and the original feature extraction network show comparable feature capture abilities.

### 4.3. Comparative Experiment of Feature Fusion Network

**Figure 9:**
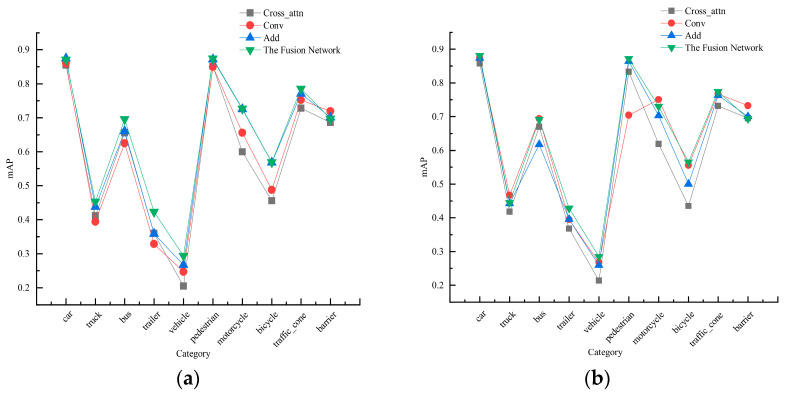
Comparisons of detection accuracy in different feature fusion networks. (**a**) Primitive feature extraction network. (**b**) EdgeNeXt_DCN feature extraction network.

Figure 9 illustrates the accuracy of networks in object detection tasks under different fusion networks and feature extraction networks. Specifically, the detection performances of various fusion networks on different categories of objects under the original feature extraction network are shown in Figure 9a. The results indicate that the multi-sensor object detection method utilizing feature fusion networks achieves average detection accuracy on nuScenes objects which is comparable to that of the original method. Notably, it shows significant improvements in detection accuracy for categories such as trucks, buses, trailers, construction vehicles, and traffic cones, with increases of 1.6%, 3.4%, 6.3%, 2.7%, and 1.5%, respectively. Compared to the cross-attention fusion network in multi-sensor detection methods, the feature fusion method shows improved detection results for various objects by 1.6%, 4.3%, 4.0%, 6.3%, 8.9%, 2.3%, 12.7%, 11.3%, 5.8%, and 1.1%, respectively. This demonstrates that the designed feature fusion network effectively promotes information interaction and feature alignment among multi-sensor features, thereby efficiently integrating image and point cloud multi-sensor features.

In the detection method employing EdgeNeXt_DCN as the image feature extraction network, the performances of various fusion networks are illustrated in Figure 9b. Compared to the original convolutional and addition fusion methods, the proposed multi-sensor fusion network exhibits superior performance in object detection. It shows improvements in detection accuracy across various categories, with percentage increases of 0.8%, 0.3%, 7.4%, 3.3%, 2.6%, 0.8%, 2.7%, 6.5%, and 1.2% and a decrease of −0.6%, respectively. Consequently, the feature fusion network exhibits robust capability in integrating features under different feature extraction networks, enhancing the accuracy of multi-sensor object detection methods.

### 4.4. Comparison Experiment of Method Performance

Table 1 presents the test results for multi-sensor 3D object detection methods using various image feature extraction and fusion networks. Specifically, Conv fusion and Add fusion refer to the two fusion methods employed by the baseline approaches.

In experiments using the Swin-Transformer as the image feature extraction network, the proposed feature fusion network improved the prediction accuracy of the object detection method. This enhancement led to better performance in detecting objects across multiple scales and in extreme scenarios. Specifically, the mAP and NDS metrics increased by 4.8% and 5.3%, respectively. Additionally, the network facilitated the alignment of multi-sensor features, reducing prediction errors in multi-sensor object detection methods. This improvement is reflected in the metrics ATE, ASE, AOE, AVE, and AAE, which decreased by 2.7%, 1.9%, 14.7%, 20.8%, and 0%, respectively, compared to the baseline method.

Replacing the Swin-Transformer in the baseline network with EdgeNeXt_DCN maintains comparable performance in object detection across various feature fusion networks. Specifically, multi-sensor object detection methods that use Conv, Add, Cross-Attention, and feature fusion modules as their fusion components outperform the Swin-Transformer. These methods show improvements in mAP of 2.8%, 0.1%, 0.4%, and −0.3%, respectively, and enhancements in the NDS of 3.8%, 0.1%, 0.3%, and 0%, respectively. Notably, the EdgeNeXt_DCN network, through its design of feature extraction and fusion networks, achieves mAP and NDS improvements of 4.5% and 5.3% over the baseline method, indicating its comparable feature-capturing capability in multi-sensor object detection. Additionally, the network’s fully convolutional architecture simplifies the training process, reduces computational costs, and facilitates the practical deployment and application of multi-sensor object detection methods.

**Figure 10:**
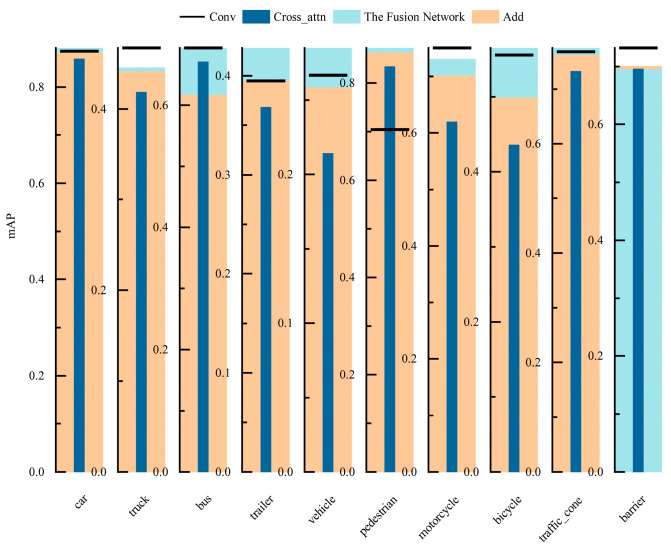
Results of object detection for each category.

Figure 10 illustrates the average detection accuracy of the designed multi-sensor object detection method across ten categories in the nuScenes dataset. The method performs exceptionally well in detecting various objects, with the exception of guardrails, where its performance surpasses the baseline network. The mAP values improved by 0.8%, 0.3%, 7.4%, 3.3%, 2.6%, 0.8%, 2.7%, 6.5%, and 1.2%, respectively, demonstrating the advantages of this multi-sensor object detection method under the BEV approach.

Through the aforementioned research, it has been established that the EdgeNeXt_DCN feature extraction network not only possesses excellent feature extraction capabilities, but also demonstrates outstanding robustness in method performance. Additionally, the proposed feature fusion network effectively integrates multi-sensor features, achieving efficient alignment across different modalities. This significantly enhances the generalization performance of multi-sensor fusion object detection methods in the BEV perspective.

**Figure 11:**
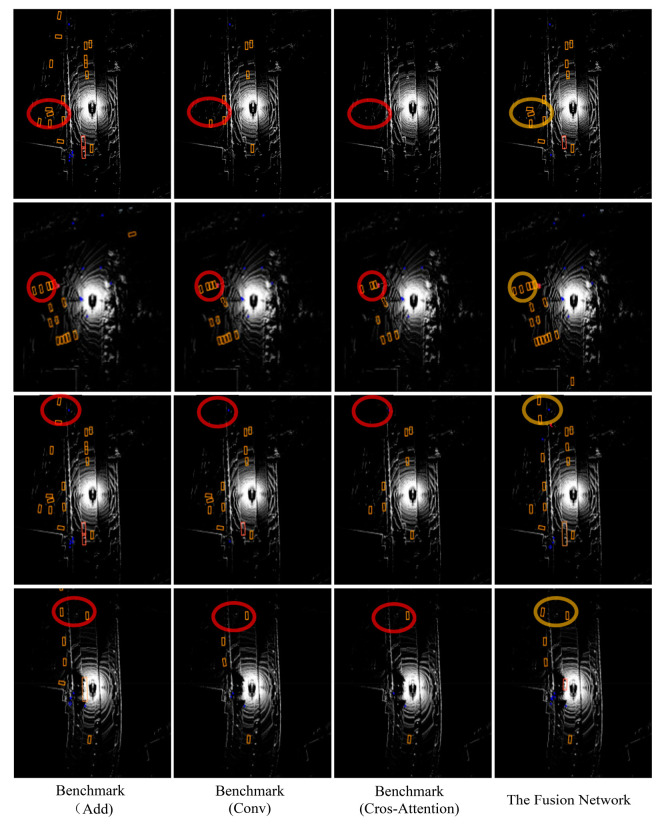
Comparison of detection results from multi-sensor fusion detection method in BEV.

The multiple method test results depicted in Figure 11 demonstrate that the baseline method utilizing the Add and Conv fusion method performs well in terms of reducing missed detections and detecting distant objects. In contrast, the baseline method employing the cross-attention mechanism does not perform as effectively in capturing subtle features of distant objects and detecting objects at a distance or partially occluded, as shown in the red circle. DeployFusion enhances the depth alignment capability between image and point cloud features while controlling computational complexity, effectively achieving precise fusion and detection of distant small features, as illustrated in the yellow circle.

**Figure 12:**
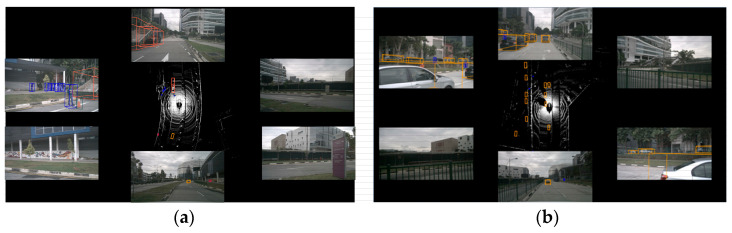
Performance of object detection in BEV of this method. (**a**) Scene 1. (**b**) Scene 2.

Figure 12 confirms the superior detection accuracy and high perception of vehicle environments achieved by the method proposed in this paper. Among them, blue boxes represent pedestrians, orange boxes represent trucks, and yellow boxes represent cars. The method can accurately identify road ends, nearby occluded areas, and vehicles in adjacent lanes, with minimal errors in object localization and size estimation, demonstrating high precision.

## 5. Model Deployment Application

### 5.1. Configuration of Mobile Computing Device

**Figure 13:**
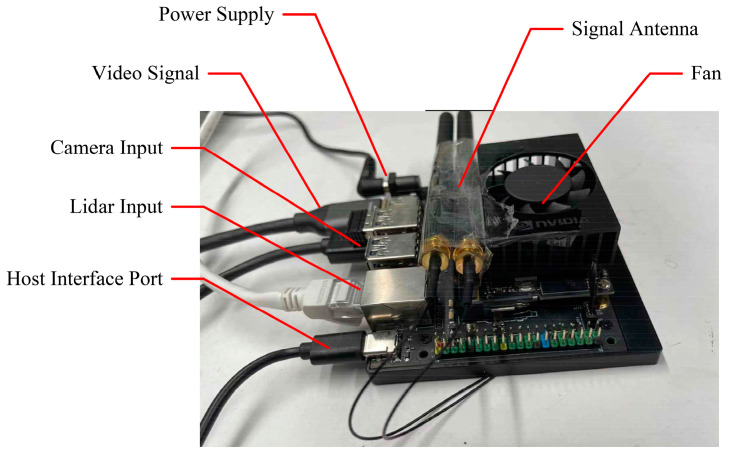
Jetson Orin NX mobile device.

The Jetson Orion NX is a hardware platform developed by NVIDIA for the application of deep learning algorithms on edge devices, suitable for small, low-power autonomous scenarios. The module offers 100 TOPS of AI performance, with specific device parameters detailed in Table 2. The important input and output locations on the Jetson Orion NX carrier board are shown in Figure 13. It features a 1024-core NVIDIA Ampere architecture GPU, equipped with 32 efficient Tensor Core computing units, capable of rapidly processing multiple tensor calculations concurrently. The powerful computational performance, rich expansion interfaces, and flexible power configuration of Jetson Orion NX make it suitable for various edge computing and deep learning application scenarios.

### 5.2. TensorRT Optimization

TensorRT is a compiler and development toolkit specifically designed for NVIDIA GPUs which automates the optimization of parallel operations in models and selects the optimal computation scheduling and parallel strategies based on the GPU architecture. It supports multiple framework inputs such as ONNX and provides Python and C++ interfaces to simplify program implementation. During the model optimization process, ONNX models are frequently used as an intermediary for weight conversion. TensorRT analyzes and optimizes the network structure and weights of the ONNX model, thereby generating a new model suitable for TensorRT inference.

**Figure 14:**
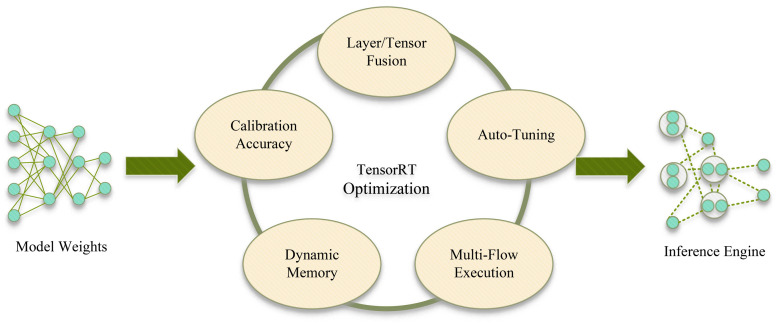
Workflow of TensorRT.

The working process of TensorRT is illustrated in Figure 14. TensorRT is compatible with multi-framework inputs such as ONNX and provides interfaces in both Python and C++. In model optimization, the ONNX model is often used as an intermediary for weight conversion. TensorRT then optimizes both the network structure and the weights of the ONNX model, generating a new model tailored for TensorRT inference. TensorRT combines techniques such as tensor fusion, memory optimization, precision calibration, multi-stream execution, and automatic core optimization to provide optimization strategies and hardware scheduling. It also features a comprehensive functional API, significantly enhancing the efficiency of deep learning inference and simplifying the model optimization and deployment process.

After the completion of network training, the convolutional parameters and normalization parameters for the weights are determined. These parameters facilitate the reduction in data access by integrating with adjacent layers, thereby enhancing computational efficiency. The common convolutional modules in the EdgeNeXt_DCN network, which include convolutional layers, batch normalization (BN) layers, and activation function layers.

**Figure 15:**
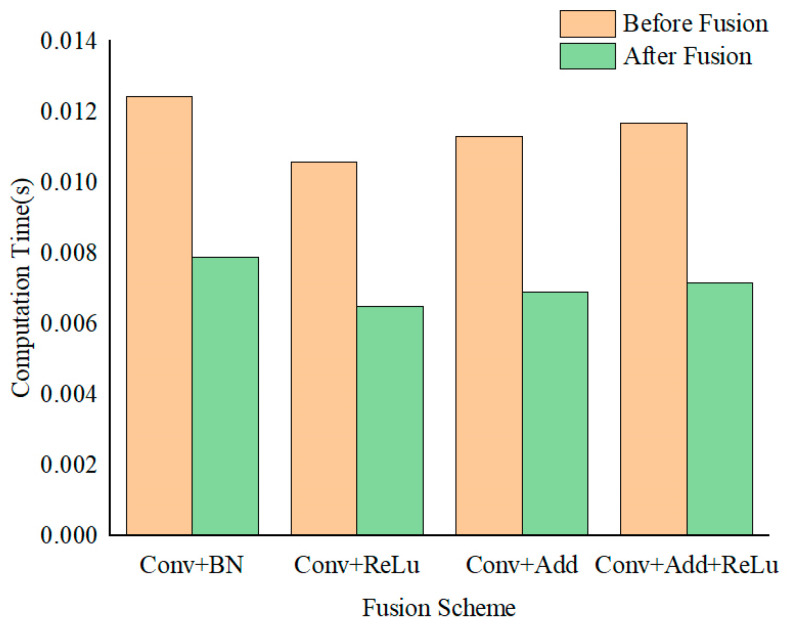
Comparison of computation time before and after operator fusion.

In the training platform, inference performance tests were conducted on convolution operators before and after fusion. The results indicate that the computational speed of various convolution operators significantly improves after fusion processing. The computational time before and after operator fusion is illustrated in Figure 15.

### 5.3. Model Testing

The multi-sensor object detection method was optimized and tested using the TensorRT toolkit. Due to the uniqueness of different network architectures, the model employed mixed-precision inference, which made it difficult to fully achieve INT8 quantization. The data preprocessing, feature extraction network, and feature fusion network of the visual branch were accelerated through FP16 and INT8 quantization using CUDA. The point cloud feature extraction, due to its specific voxelization and sparse convolution implementation, relied on CUDA parallel computing for acceleration. Finally, the detection network and post-processing of partial prediction results were optimized by combining TensorRT with CUDA multi-core parallel computing technology.

**Figure 16:**
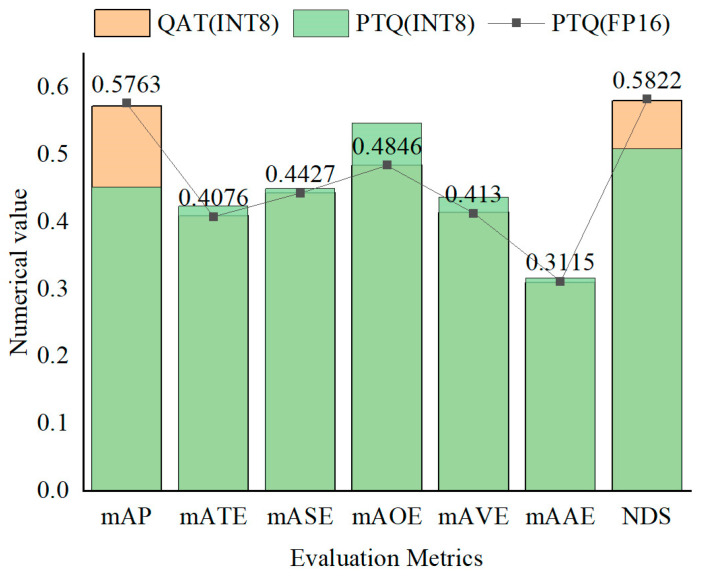
Comparison of detection methods in various quantifiers and accuracy levels.

As shown in Figure 16, the performance of the multi-sensor fusion detection method varies under different quantization methods and precisions. In post-training quantization (PTQ), the model with FP16 precision achieves higher mAP and NDS scores by 0.12 and 0.07, respectively, compared to the INT8 precision model. Quantization-aware training (QAT) can optimize performance by adjusting network weights; with INT8 precision, QAT-quantized models outperform PTQ-quantized models in terms of mAP, NDS, and error metrics, with improvements of 0.12 and 0.07. Although the QAT (INT8) model is similar to the PTQ (FP16) model in detection performance, QAT operations are more complex and difficult to implement. In contrast, PTQ is simpler to implement but may result in precision loss.

**Figure 17:**
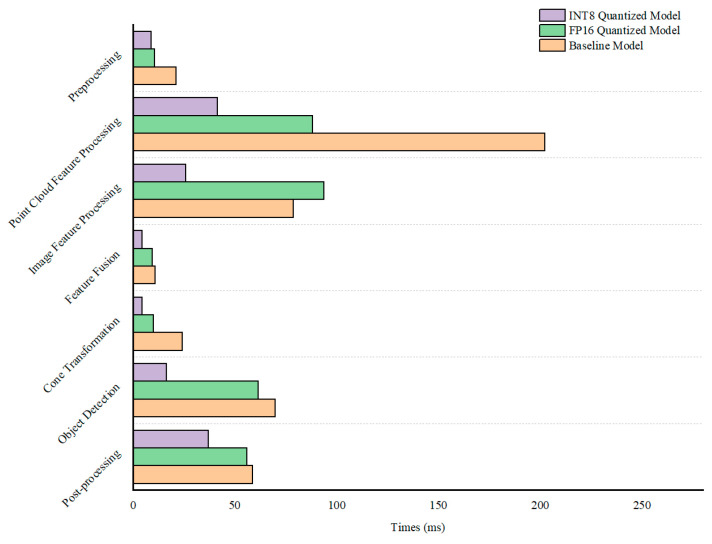
Comparison of inference time before and after model quantification in detection.

In terms of methodic inference latency, the test analysis of the multi-sensor fusion detection method before and after quantization is shown in Figure 17. The figure compares the inference latency of the original model under the PyTorch framework with that of the model quantized using the TensorRT tool for INT8 and FP16. Through INT8 type quantization, the performance was significantly enhanced. The inference latency for point cloud feature processing decreased by 79.5%; image feature extraction latency by 67.1%; and the latencies for feature fusion, frustum conversion, and object detection network by 11.7%, 57.9%, and 11.9%, respectively. The overall inference latency was reduced to 138.74 ms, with the frame rate increasing to approximately 7 frames per second, resulting in a substantial improvement in computational efficiency.

In the FP16 quantization experiments, the inference latency of the image feature extraction network decreased by 9.1%. Examining the diagram from top to bottom, the inference latency for each component was reduced by 56%, 11.7%, 57.9%, 11.9%, and 4.5%, respectively. The total latency was approximately 220.04 milliseconds, and the frame rate was about 5 frames per second. Compared to INT8, FP16 occupies more memory, consumes more program access, and has lower inference computational efficiency. As shown in Figure 17, the “INT8 Quantization Model” and “FP16 Quantization Model” columns indicate that the inference latency of the FP16 quantization model is significantly higher than that of the INT8 quantization model.

**Figure 18:**
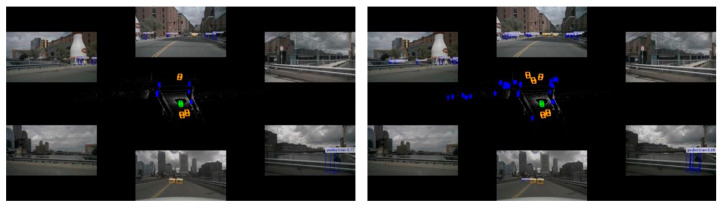
Detection result of method on mobile devices.

The detection performance of the method deployed on edge devices is illustrated in Figure 18, with the upper part showing INT8 quantization and the lower part showing FP16 quantization. Among them, blue boxes represent pedestrians, yellow boxes represent cars, and green represents the ego vehicle. Due to the network’s sensitivity to parameter precision in method output, the full network quantization results of INT8 quantization exhibit more missed detections compared to FP16 quantization. Specifically, FP16 quantization fails to detect vehicles at a distance and misses multiple pedestrians in the front and front-left views. However, in the rear and rear-right views, the detection of nearby vehicles and pedestrians performs relatively better. Given the variability in deployment environments, our next step is to plan the migration of our research results to the more affordable Jetson Nano device for testing.

## 6. Conclusions

This paper addresses the field of 3D object detection for autonomous driving, with our work aiming to enhance the accuracy of monocular 3D object detection by effectively fusing information from LiDAR and camera modalities. In this paper, a deployable 3D object detection framework called DeployFusion is proposed, which integrates multi-sensor information under the BEV. Firstly, DeployFusion propose a lightweight and efficient image feature extraction network called EdgeNeXt_DCN, which integrates residual branches to prevent degradation in deep networks and employs deformable convolutions to increase the receptive field while reducing computational load, achieving feature learning capabilities comparable to Swin-Transformer. To accurately achieve long-range detection, we construct a dual-stage fusion network that aligns image features with point cloud features, supplements environmental information, and optimizes the fusion and alignment of features. Moreover, compared to traditional attention mechanisms, the transpose attention used significantly reduces computational complexity, making it suitable for deployment on mobile computing devices. Experiments show that our method improves the NuScenes detection score and the average precision of objects by 4.5% and 5.5%, respectively, compared to the baseline model network.

Time series information allows for the acquisition of historical and future temporal contexts of an object, positively impacting the model’s ability to grasp trend changes at various time points and predict future object position and state. Modeling temporal context helps to address challenges posed by object deformation, occlusion, and other external factors. Next, we will further optimize the use of time-series information to tackle instance depth estimation.

## Figures and Tables

**Table 1:** Experimental results of multi-sensor object detection method.

Feature Fusion Network	Fusion Network	mAP↑	ATE	ASE	AOE	AVE	AAE	NDS
Swin-Transformer	Conv	0.592	0.320	0.277	0.564	0.412	0.194	0.619
	Add	0.624	0.309	0.262	0.424	0.347	0.192	0.659
	Cross-Attention	0.581	0.309	0.263	0.434	0.356	0.193	0.635
	Feature Fusion Network	**0.640**	**0.293**	**0.258**	**0.417**	**0.294**	**0.194**	**0.674**
EdgeNeXt_DCN	Conv	0.620	0.307	0.264	0.426	0.344	0.189	0.657
	Add	0.625	0.297	0.261	0.413	0.357	0.201	0.660
	Cross-Attention	0.584	0.311	0.261	0.427	0.351	0.194	0.638
	Feature Fusion Network	**0.637**	**0.293**	**0.256**	**0.418**	**0.289**	**0.191**	**0.674**
ResNet50	Conv	0.576	0.349	0.296	0.634	0.976	0.245	0.538
	Feature Fusion Network	**0.600**	**0.315**	**0.268**	**0.468**	**0.438**	**0.188**	**0.632**

**Table 2:** Jetson Orin NX parameter table.

Parameter	Number	Unit
GPU Architecture	Ampere	
AI Performance	100	TOPS
GPU Maximum Frequency	918	MHz
CUDA Core	1024	
Tensor Core	32	
Memory	16	GB
Bandwidth	102.4	GB/s
CPU	Arm^®^ Cortex-A78AE	
CPU Core	8	
CPU Maximum Frequency	2	GHz
DL Accelerator	2	
DLA Maximum Frequency	614	MHz

## Data Availability

Data are contained within the article.
